# Role of tyrosine kinases in bladder cancer progression: an overview

**DOI:** 10.1186/s12964-020-00625-7

**Published:** 2020-08-14

**Authors:** Amir Sadra Zangouei, Amir Hossein Barjasteh, Hamid Reza Rahimi, Majid Mojarrad, Meysam Moghbeli

**Affiliations:** 1 Student Research Committee, Faculty of Medicine, Mashhad University of Medical Sciences, Mashhad, Iran; 2 Department of Medical Genetics and Molecular Medicine, School of Medicine, Mashhad University of Medical Sciences, Mashhad, Iran

**Keywords:** Bladder cancer, Tyrosine-kinase, Diagnosis, Targeted therapy, Panel marker

## Abstract

**Background:**

Bladder cancer (BCa) is a frequent urothelial malignancy with a high ratio of morbidity and mortality. Various genetic and environmental factors are involved in BCa progression. Since, majority of BCa cases are diagnosed after macroscopic clinical symptoms, it is required to find efficient markers for the early detection. Receptor tyrosine-kinases (RTKs) and non-receptor tyrosine-kinases (nRTKs) have pivotal roles in various cellular processes such as growth, migration, differentiation, and metabolism through different signaling pathways. Tyrosine-kinase deregulations are observed during tumor progressions via mutations, amplification, and chromosomal abnormalities which introduces these factors as important candidates of anti-cancer therapies.

**Main body:**

For the first time in present review we have summarized all of the reported tyrosine-kinases which have been significantly associated with the clinicopathological features of BCa patients.

**Conclusions:**

This review highlights the importance of tyrosine-kinases as critical markers in early detection and therapeutic purposes among BCa patients and clarifies the molecular biology of tyrosine-kinases during BCa progression and metastasis.

Video abstract

## Background

Bladder cancer (BCa) is the 10th most frequent malignancy worldwide [1], and the fourth most prevalent malignancy among American males with 7% of all cancer cases [2]. There are an estimated 62,100 new BCa cases and 13,050 deaths annually among American males [2]. Approximately 430,000 new cases of BCa and 150,000 deaths were reported in 2012 worldwide [3]. The highest incidence rates were observed in both sexes in European countries and Northern America, while the Lebanese females had the highest rates in the world [1]. There is a correlation between age and higher risk of BCa progression [4]. The BCa is also three to four times more frequent among males compared with females [5]. The prevalence of BCa can be associated with environmental and genetic determinants, which are different among racial/ethnic groups [6, 7]. Based on histopathologic features of tumor cells, there are three main types of BCa including**:** transitional cell carcinoma, squamous cell carcinoma, and adenocarcinoma, which account for 90, 5%, and less than 2% of BCa cases, respectively [8]. Non-muscle-invasive bladder cancer (NMIBC) or superficial bladder cancer which constitutes about 75% of newly diagnosed cases is confined to the lamina propria without invasion to underlying muscle tissues. Muscle-invasive bladder cancer (MIBC) involves about 25% of cases that has invasion to the muscularis propria and perivesical fat [9]. There are various treatment options for the BCa including radical cystectomy, transurethral resection, chemotherapy, radiotherapy, immunotherapy, and targeted therapies against specific signaling proteins [10, 11]. Over the past decades, mutations in kinases have been shown to be engaged in bladder malignancies [12]. Protein kinases are a large family of proteins with more than 500 members which are encoded by ~ 2% of all human genome. Their main function is the phosphorylation of definite amino acids on key proteins involved in critical cellular mechanisms such as proliferation, differentiation, survival, and apoptosis [13, 14]. As these cellular processes are of paramount importance, the enzymatic function of these kinases is strictly regulated [15]. Tyrosine-kinases (TKs) are one of the major types of protein kinases which induce phosphorylation of tyrosine residues on a substrate protein. TKs are found in nuclear and membrane-bound forms as well as transmembrane receptors [16]. There are about ninety TKs in human genome which are involved in various cellular processes such as differentiation, metabolism, motility, and proliferation [17, 18]. Receptor tyrosine-kinases are up regulated in various tumors and are considered to be potential oncogenes involved in cancer initiation and development [19, 20]. Therefore, targeting these oncogenes and inhibiting their expression may result in good clinical outcomes [21]. The majority of TKs share a similar structure consisting of a conserved catalytic domain and regulatory domains which are located within or outside the catalytic domain. The catalytic domain contains 250–300 amino acids in 12 conserved subdomains and catalyzes the transfer of a phosphate group from ATP to the tyrosine residue. Activation and recruitment of downstream signaling pathways occur following this phosphorylation. Regulatory domains regulate the kinase activity and its localization in response to different stimuli [22, 23]. Studies have shown oncogenic characteristics of TKs [24]. Dysregulation of TKs by gain of function mutations or over expression occurs in various malignancies like BCa which accelerates tumor proliferation and progression [25, 26]. Therefore, inhibiting signaling pathways of different tyrosine kinases using tyrosine-kinase inhibitors (TKIs) have been reported as efficient method of tumor targeted therapies [27–29]. BCa patients are diagnosed with a broad range of tumor behaviors from low grade and stage tumors with less aggressiveness to tumors with advanced grade, stage, and distant metastasis. Clinicopathological features are commonly used to predict tumor growth, recurrence, and patient’s survival. However, the gene expression profiling and molecular pathway analysis have gained growing attention as a novel and promising methods for the prediction of disease course and prognosis in BCa patients [7, 30, 31]. Since, tyrosine-kinases have essential roles in BCa progression, in present review we have summarized all of the studies which have been assessed the role of tyrosine-kinases in BCa patients in the world (Table 1).

**Table 1:** All of the tyrosine kinases which have been significantly associated with clinicopathological features of BCa patients in the world

Study	Year	Gene	Country	Sample	Results
RTK class I					
COOMBS [32]	1993	HER2	UK	91 tumor tissues 99 control tissues	Correlation between tumor grade and HER2 up regulation
SIMON [33]	2003	HER2	Switzerland	2317 tumor tissues	Correlation between tumor grade and HER2 up regulation
NADOUSHAN [34]	2009	HER2	Iran	75 patients	Correlation between tumor grade and HER2 up regulation
Krüger [35]	2002	HER2	Germany	138 patients	Correlation between tumor grade and HER2 up regulation
COOGAN [36]	2004	HER2	USA	54 patients	Correlation between tumor grade and HER2 up regulation
LATIF [37]	2004	HER2	UK	75 patients	Increased HER2 copy number
LATIF [38]	2003	HER2	UK	25 patients	Increased HER2 copy number
FLEISCHMANN [39]	2011	HER2	Switzerland	150 patients	HER2 amplification was correlated with poor prognosis.
HANSEL [40]	2008	HER2	USA	53 patients	HER2 amplification was correlated with high-stage (pT4).
EISSA [41]	2005	HER2	Egypt	88 patients	HER2 expression was correlated with lymph node involvement.
ABDELRAHMAN [42]	2019	HER2	Egypt	60 patients	HER2 up regulation was correlated RFS and PFS.
DING [43]	2015	HER2	China	238 patients	HER2 up regulation was correlated with advanced grade and stage.
BREYER [44]	2016	HER2	Germany	355 patients	HER2 up regulation was correlated with advanced grade and stage.
ARIKAN [45]	2015	HER2	Turkey	44 patients 40 controls	HER2 urinary level was correlated with advanced grade.
KOLLA [46]	2008	HER2	India	90 patients	HER2 expression was correlated with advanced grade, stage, and lymph node metastasis.
KOGA [47]	2011	HER2	Japan	35 patients	HER2 up regulation was correlated with poor prognosis.
INOUE [48]	2014	HER2	Japan	201 patients	HER2 up regulation was correlated with CRT resistance.
SASAKI [49]	2014	HER2	Japan	171 patients	HER2 up regulation was correlated with grade and *recurrence.*
KIM [50]	2016	HER2	South Korea	6 patients	HER2 protein up regulation and gene amplification
MEMON [51]	2004	HER3, HER4	Denmark	88 patients	HER3 and HER4 up regulations were correlated with good prognosis.
GUNES [52]	2013	EGFR, HER2, ErbB3, ErbB4	Turkey	40 patients	EGFR and HER2 were down regulated and up regulated respectively.
Türkeri [53]	1998	EGFR	Turkey	21 patients	EGFR expression was correlated with stage.
POPOV [54]	2004	EGFR	France	113 patients 10 controls	EGFR expression was correlated with survival.
ARFAOUI [55]	2016	EGFR	Tunisia	64 patients 6 controls	EGFR expression was correlated with grade and stage.
BERGER [56]	1987	EGFR	UK	31 patients	EGFR expression was correlated with grade and stage.
KHALED [57]	2009	EGFR	Egypt	59 patients	EGFR expression was correlated with stage and survival.
LIPPONEN [58]	1994	EGFR, HER2	Finland	234 patients	EGFR expression was correlated with grade and aneuploidy.
MEMON [59]	2006	EGFR, HER2, HER3, HER4	Denmark	88 patients	HER1–4 were correlated with poor prognosis.
KIM [60]	2014	EGFR	Korea	80 patients	EGFR up regulation was correlated with chemo resistance.
COLQUHOUN [61]	2006	EGFR	UK	110 patients	EGFR expression was correlated with recurrence.
Thøgersen [62]	1999	EGFR	Denmark	54 patients	EGFR up regulation was correlated with stage.
MANSOUR [63]	2018	EGFR	Egypt	58 patients	EGFR up regulation was correlated with poor prognosis.
Bryan [64]	2015	EGFR	UK	436 patients 60 controls	EGFR urine levels were correlated with grade and survival.
CHU [65]	2013	EGFR	China	908 patients 1239 controls	Polymorphism was correlated with BCa progression.
MASON [66]	2009	EGFR	USA	857 patients 1191 controls	Polymorphism was correlated with BCa progression.
LI [67]	2015	HER2, EGFR, VEGFR	USA	16 patients	EGFR up regulation
RAJJAYABUN [63, 68]	2005	EGFR, HER2	UK	58 patients	HER2 and EGFR up regulations
IMAI [69]	1995	EGFR, HER2	Japan	30 patients	HER2 and EGFR up regulations were correlated with recurrence.
LI [70]	2018	EGFR, HER2	China	56 patients 10 controls	HER2 and EGFR expressions were correlated with stage.
KASSOUF [71]	2008	EGFR, HER2, ErbB-3, ErbB-4	Canada	248 tumors	EGFR and ErbB-4 expressions were correlated with grade and survival.
RTK class II					
GONZALEZ-ROBIN [72]	2014	IGF1R	USA	100 patients	IGF1R expression was correlated with pT classification.
ROCHESTER [73]	2007	IGF1R	UK	40 tumor tissues 15 control tissues	IGF1R expression was correlated with pT classification.
Metalli [74]	2010	IGF1R	USA	5 patients	IGF1R up regulation
Iozzo [75]	2011	IGF1R	USA	40 patients	IGF1R up regulation
RTK class V					
MARZIONI [76]	2009	FGFRI, FGFR2	Italy	17 patients	FGFRI, FGFR2, and bFGF up regulations
Dueñas [77]	2015	PIK3CA, FGFR3	Spain	87 patients	PIK3CA and FGFR3 mutations were correlated with grade and recurrence.
JUANPERE [78]	2012	FGFR3, PIK3CA, KRAS, AKT1,	Spain	88 patients	Mutations were correlated with grade.
KOMPIER [79]	2010	FGFR3, HRAS, KRAS, NRAS, PIK3CA	Netherlands	257 patients	Mutations were correlated with grade.
PANDITH [80]	2016	FGFR3	India	65 patients	Mutation was correlated with grade and stage.
BERTZ [81]	2014	FGFR3	Germany	61 patients	Mutation was correlated with prognosis and recurrence.
BILLEREY [82]	2013	FGFR3	France	132 patients	FGFR3 mutation was correlated with pT classification.
VAN OERS [83]	2007	FGFR3	Germany	255 patients	FGFR3 mutation was correlated with grade.
VAN OERS [84]	2009	FGFR3	UK, France	280 patients	FGFR3 mutation was correlated with pT classification and prognosis.
MHAWECH-FAUCEGLIA [85]	2006	FGFR3	USA	254 patients	FGFR3 up regulation was correlated with grade and stage.
BREYER [86]	2018	FGFR3	Germany	296 patients	FGFR3 expression was correlated with grade.
BODOOR [87]	2010	FGFR3	Jordan	130 patients	FGFR3 expression was correlated with grade and stage.
PEDREGOSA [88]	2017	FGFR3	Spain	55 patients	FGFR3 expression was correlated with tumor size and survival.
YANG [89]	2018	FGFR3	China	52 patients	FGFR3 mutation was associated with better prognosis.
ABDUL-MAKSOUD [90]	2016	FGFR1	Egypt	80 patients 80 controls	FGFR1 expression was correlated with grade and stage.
RTK classes of VIII, IX, and XIII					
MCNEIL [91]	2014	c-MET	USA	183 patients	Urinary MET levels were correlated with muscle invasion.
KIM [92]	2015	c-MET, AXL, PDGFR	Korea	165 patients 34 controls	Expressions were correlated with prognosis.
CHENG [93]	2005	c-MET	Taiwan	183 patients	RON and MET expressions were correlated with survival.
Abraham [94]	2006	EphA2	USA	64 tumor tissues 13 normal tissues	EphA2 expression was correlated with tumor stage.
XIA [95]	2006	EphB4	USA	15 patients	EphB4 up regulation
Xie [96]	2017	DDR1	China	44 patients	DDR1 expression was correlated with poor prognosis.
Tsai [97]	2016	DDR2	Taiwan	340 patients	DDR2 up regulation was correlated with poor prognosis, infiltrative pattern, higher grade, advanced T stage, and metastatic status.
Other tyrosine kinase receptors					
LAI [98]	2010	BDNF, TrkB	Taiwan	116 tumor tissues	BDNF and TrkB up regulations
PAN [99]	2005	c-KIT	USA	52 patients	c-KIT up regulation
SHAMS [100]	2013	c-KIT	Egypt	120 patients	c-KIT up regulation
Non-receptor tyrosine kinases					
ASAI [101]	2018	FES	Japan	203 patients	FES up regulation was correlated with poor prognosis.
NOMURA [102]	2015	DYRK2	Japan	44 patients	DYRK2 expression was correlated with survival.
GUO [103]	2011	ETK	USA	233 patients	ETK up regulation was correlated with grade and prognosis.

## Main text

### RTK class I

The ERBB family includes ERBB1 (EGFR), ERBB2 (HER2), ERBB3 (HER3), and ERBB4 (HER4) that are the class I receptors of tyrosine-kinases (RTKs). The HER2/neu is involved in transduction of mitogen signals through activation of various signaling pathways such as MAP kinase, PI3 kinase, and MYC [104, 105]. The prevalence of HER2/neu over expression in BCa is one of the most elevated among human malignancies, with a range of 9 to 34% of examined cases [106, 107]. HER2 up regulation is mainly due to gene amplification which triggers intracellular pathways that promote cell proliferation, migration, and aggressiveness of tumor cells [105, 108]. Various studies have been reported that there were significant associations between tumor grade and HER-2 up regulation or gene amplification among different population of BCa patients [32–36]. The pT2 tumors have a considerable amount of mutations compared with pTa/Ti tumors [109]. Protein up regulation and gene amplification of HER2/neu occur more frequently in pT2 tumors in comparison with pTa/Ti tumors which are correlated with a poor prognosis [107, 110–114]. A significant high frequency of chromosome 17 polysomy (97%) and increased HER2/neu copy number (92%) were also observed in a sample of BCa patients. Polysomy 17 and HER2/neu up regulation were frequent in G3 pT2 tumors [37]. Another study has been shown that there was HER2/neu abnormality in pT2 BCa tumors before muscle invasion. Polysomy 17 and HER2/neu amplification and up regulation were correlated with advanced disease. Therefore, the HER2/neu deregulation was observed before the muscle invasion [38]. A quarter of patients experiencing cystectomy and lymphadenectomy for N0M0 staged BCa showed lymph node metastases which resulted in death among two thirds of patients [115, 116]. The chemotherapy is not efficient in metastasizing bladder malignancy, and new therapeutic modalities are required [117, 118]. It has been observed that the HER2 amplification was significantly increased in urothelial bladder tumors with lymph node metastasis compared with initial tumors. HER2 amplification was also significantly associated with poor prognosis [39]. Another study has been shown that the patients with distant metastasis had co amplifications of HER2 and MYC in a sample of BCa cases. There was also a significant correlation between HER2 or MYC amplification and high-stage (pT4) tumor [40]. It has been shown that there were significant increased levels of HER2/neu expression in a sample of malignant BCa patients compared with benign and healthy cases. There were significant correlations between HER2/neu, ploidy, SPF, and lymph node involvement [41].

About 75% of BCa patients have NMIBC that can be limited to the mucosa (Ta) or carcinoma in situ (Tis) without stromal invasion or submucosal invasion (T1). Unsaturated fat synthase (FASN) down regulation induces apoptosis and represses tumor progression and metastasis [119]. FASN can be phosphorylated by mTOR and HER2/neu which is associated with its function and cellular localization [120]. HER2/neu triggers PI3K/AKT and Ras/Raf/MAPK signaling pathways as the stimulators of FASN. It has been observed that there were significant correlations between FASN expression, tumor size, grade, recurrence, and stage. Moreover, there was a correlation between FASN and HER2/neu which had prognostic value among NMIBC patients. HER2/neu up regulated the FASN through PI3K and MAPK pathways. FASN and HER2/neu up regulations were associated with shorter RFS and poor PFS among NMIBC cases [42]. HER2 up regulation has been observed in a sample of NMIBC patients which was significantly correlated with advanced grade and stage, bigger size, and adjacent tissues invasion. HER2 positive patients had markedly lower progression-free survival in comparison with HER2 negative cases [43]. It has been reported that the over expression of ERBB2 was significantly correlated with high grade and advanced stage tumor among a group of NMIBC patients [44]. A significantly higher level of urinary HER2/neu was also observed among a sample of NMIBC patients compared with normal subjects. Moreover, HER2/neu level/urinary creatinine ratio was significantly associated with advanced tumor grade [45]. It has been reported that the patients with positive HER2 expression had significantly shorter disease-free survival compared with negative HER2 expression cases in a sample of MIBC patients. This difference was more significant in patients with advanced stage and grade who were candidates for adjuvant treatment. The HER2/neu expression was significantly associated with higher tumor grade and stage, and lymph nodes involvement. Moreover, patients with HER2 up regulation had a worse disease-specific survival compared with normal HER2 expressed patients [46]. It has been observed that the HER2 and NFkB up regulations had a key role in tumor cells resistance against chemotherapy among a sample of MIBC patients. HER2 and NFkB Over expressions were significantly correlated with poorer survival in chemotherapeutic–treated MIBC patients who were undergone cystectomy. High expression levels of NFkB were also significantly associated with drug resistance in MIBC patients. Therefore, HER2 and NFkB up regulations can be resulted in a worse prognosis in chemotherapeutic–treated MIBC patients who were undergone cystectomy [47]. Another study has been shown that there was a significant association between HER2 up regulation and CRT resistance. Targeting HER2 improves prognosis of MIBC cases that were treated with CRT-based bladder-sparing methods [48].

Upper urinary tract urothelial carcinoma (UUTUC) is an uncommon type with frequency of 5–10% among all urothelial carcinomas. It has been shown that there was significant association between HER2 protein up regulation, gene amplification, grade, and *shorter recurrence period*. HER2 positivity was more in patients over 70 years old compared with patients under 70 years old [49]. Plasmacytoid urothelial carcinoma is also another rare type of urothelial carcinoma that is distinguished by plasma-cell-like cancer cells [121, 122]. It has been reported that the plasmacytoid urothelial carcinoma patients had HER2 protein up regulation and gene amplification [50]. Heregulin (HRG) is ligand of ERBB family of receptors that can regulate cell proliferation, apoptosis, and differentiation [123–125]. It has been reported that the HER3 and HER4 up regulations and their initiating ligands are correlated with good prognosis among BCa patients. Patients with high HER3 and HER4 expressions had increased survival rate. There was significant HRG2b loss in invasive tumors in comparison with non-invasive tumors. Moreover, there was a significant association between (HER3 and HER4) and (HRG2 and HRG4) expressions [51]. It has been show that there were significant different levels of ErbB4 protein expressions between a sample of bladder tumors and normal margins. EGFR and HER2 were down regulated and up regulated respectively in tumor tissues in comparison with non-malignant bladder tissues [52].

EGFR functions in a dimeric structure of different EGF receptors for the signal transduction [126, 127]. Dimeric pairs are related to the concentration of both receptors and specific ligands and also the affinities between receptors [128, 129]. It has been reported that there were associations between the elevated levels of growth factors and also their receptors and tumor recurrence in a sample of BCa patients. Tumor tissues had increased levels of EGFR and related growth factors at early stages [53]. Another study has been reported that there was EGFR up regulation in invasive BCa tumors. Progression free survival rate in patients without any progression were significantly lower among EGFR positive cases [54]. It has been reported that there were associations between EGFR expression, tumor grade, and stage [55, 56]. The increased EGFR levels of expressions were significantly associated with advanced stage and overall survival rates among muscle invasive bilharzial bladder cancer (MI-BBC) patients [57]. Another study has been shown that the EGFR up regulation was significantly correlated with aneuploidy and polysomy in a sample of BCa tumors. A significant association was also observed between high expression of EGFR and P53. Moreover, up regulation was positively associated with tumor invasiveness, aneuploidy, non-papillary type, and grade. Therefore, highly expressed EGFR is typically a delayed event during BCa progression due to genomic instability [58]. EGF and five other ligands capable of binding to EGFR are among the various family-specific EGF ligands, while heregulins are the HER3 and HER4 ligands [130]. The expression levels of HER1–4 were assessed in bladder tumors, which showed that the up regulation of HER3 and/or HER4 was a protective factor against the negative outcome of HER1 and/or HER2 over expression. Over expression of either HER1 or HER2 in HER3 and HER4 down regulated tumors were correlated with decreased survival. Therefore, HER1 and HER2 over expressions resulted in poor prognosis and tumor progression only if there were HER3 and HER4 under expressions. Patients with high expression levels of HER1 but HER3 and HER4 down regulations had a poorer prognosis than those with high levels of HER1, HER3, and HER4. Furthermore, patients expressing high levels of HER2 but HER3 and HER4 down regulations had a significantly lower survival rate compared with patients with up regulated HER2, HER3, and HER4 [59].

Radical cystectomy is the standard treatment option for MIBC, however about half of these cases had tumor metastasis during 2 years [131, 132]. Patients with metastatic BCa receive systemic Cisplatin-based therapy as the first-line treatment modality, which has a poor prognosis in advanced tumor stage [133, 134]. It has been reported that the EGFR up regulation was correlated with chemo resistance in a sample of BCa patients. The S100A9 and EGFR suppressions decreased tumor cells viability and Cisplatin-resistance. Moreover, the S100A9 and EGFR up regulations were observed in MIBC patients [60]. Another study has been reported that there was a significant correlation between EGFR negative tumor and positive radio therapeutic response at 3-month check cystoscopy. Lack of radiotherapy response at 3-month check cystoscopy was an autonomous prognostic indicator for the reduced survival rate of bladder malignancy. Positive EGFR status predicted future local relapse following a previous complete radio therapeutic response [61]. It has been shown that there was EGFR up regulation in muscle invasive in comparison with lower invasive bladder tumors which were also correlated with advanced tumor stage. EGFR and TGF-α co-expression was significantly correlated with muscle invasion [62]. The EGFR up regulation can predict a poor prognosis compared with lack of EGFR expression in a sub population of MIBC patients. There was also a significant association between EGFR over expression and tumor relapse after adjuvant chemotherapy for advanced BCa, which introduced EGFR as a useful prognostic marker of BCa [63]. It has been shown that some high-grade bladder tumor patients had increased urinary level of EGFR. EGFR levels in urine can be used as a predictor of survival [64]. The EGFR 3′UTR 774 T > C polymorphism can be associated with higher risk of BCa progression. Patients with EGFR 774CC genotype were significantly more susceptible to BCa in comparison with 774TT/TC genotype [65]. There was also a significant correlation regarding EGFR_03 and EGFR_05 variants with a higher risk of BCa progression, and EGFR_05 and EGFR_1808 variants with a prolonged survival. EGFR_03 and EGFR_05 polymorphisms were correlated with higher BCa risk. Patients with polymorphic EGFR alleles showed higher survival rate than patients with wild-type EGFR. However, mutated type of the EGF_04 ligand showed lower survival rate than wild-type [66].

Micropapillary urothelial carcinoma (MPUC) is an aggressive urothelial cancer with poor prognosis which is due to the high tendency of tumor for lymphovascular invasion. Majority of the MPUC patients have a high grade and stage tumor at the time of diagnosis [135]. It has been shown that there was a significant EGFR up regulation in a sample of MPUC patients [67]. Tumors with muscle invasion (stage T2-T4) showed a significant higher progression and metastasis, with decreased 5-year survival rates. Cell-to-cell and cell-to-matrix signals are mainly transmitted through tyrosine-kinases that results in alteration of cell differentiation, motility, attachment, and apoptosis [17]. It has been reported that there were HER2 and EGFR up regulations in a sample of BCa in comparison with typical urothelium. The HER2 and EGFR over expressions had an important role in the early stages of tumor progression. Moreover, there were significant correlations between HER2 expression and HER2 and EGFR coexpression in patients with T1 disease [68]. It has been reported that there was a significant association between nuclear positivity of c-MYC and HER2 up regulation among a group of transitional cell BCa patients. Cytoplasmic c-MYC expression was also correlated with grade, papillary status, and EGFR/HER2 up regulation [136]. EGFR- and/or HER2 up regulation has been also reported to have a significant association with higher frequency of recurrence in BCa patients [69]. It has been observed that there was a significant positive association between HER2 and EGFR expressions in a sub population of transitional BCa patients. Bladder tumor cells had significantly higher levels of EGFR and HER2 expressions compared with controls. Patients with advanced tumor grade and stage, and tumor relapse had significant increased expression of EGFR in comparison with patients with lower grade and stage of tumor, and lack of recurrence. Patients with advance stages and tumor relapse also showed increased levels of HER2 expression compared with recurrence free patients with lower tumor stages [70]. It has been shown that the EGFR up regulation and ErbB4 down regulation were significantly correlated with tumor aggressiveness, advance grade, and poor overall survival in a sub population of BCa patients. The patients with EGFR down regulation had higher recurrence-free survival in comparison with patients with EGFR up regulation. Patients without ErbB4 down regulation had better 5-year overall survival and were more likely to have small, low-grade, and non-invasive tumors [71].

### RTK class II

Insulin-like growth factor-1 receptor (IGF1R) is belonged to the class II RTKs which have pivotal functions in regulation of cell proliferation, migration, apoptosis, and differentiation. IGF-IR has an anti-apoptotic function during tumor progression [137]. It has been shown that the IGF1R expression was correlated with the race and pT classification in malignant UC cases. There was a negative association between pT classification and IGF1R expression in which the IGF1R over expression was less in pT4 cases compared with lower pT categories. IGF1R over expression was significantly correlated with mortality which can be introduced as a prognostic factor of BCa [72]. Another study has been reported that there was significant IGF1R over expression in a sample of BCa tissues compared with healthy urothelium. Both invasive and superficial (Ta–T1) bladder tumors had higher expression levels of IGF1R compared with normal bladder tissue [73]. It has been observed that the IGF-IR promotes bladder tumor cells migration and invasion through AKT-ERK related activation of Paxillin. Since, phosphorylated Paxillin colocalizes with FAK in focal adhesion in migrating cells, Paxillin suppression reduced the invasion of 5637 and T24 cell lines. There was also IGF-IR up regulation in a sample of invasive BCa tissues compared with normal margins [74]. Decorin is belonged to the small leucine-rich proteoglycans involved in tumor progression via RTKs suppression [75, 138]. It has been shown that there were significant IGF-IR over expression in high grade in comparison with low grade BCa samples. There was also an inverse correlation between the levels of decorin and IGF-IR expressions in BCa. Moreover, Decorin suppressed tumor cell invasion through IGF-IR inhibition [139].

### RTK class V

Basic Fibroblast Growth Factor (bFGF) is a cationic protein that binds with heparin and is involved in angiogenesis and tumor progression. The basic FGFR is belonged to the class V of RTKs, and syndecans are essential for its activation. Ligand binding causes FGFR dimerization, which leads to the kinase domain autophosphorylation and phosphorylation of effector signaling proteins. Syndecans binding with both bFGF and their FGFRs functions as stimulators while syndecans that bind only bFGF operate as signaling inhibitors. It has been shown that there were significant FGFRI, FGFR2, bFGF, and syndecan1–4 up regulations in a sample of BCa tissues [76]. Different changes and variations have been detected in the members of phosphatidylinositol 3-kinase (PI3K) pathway in urinary BCa [140, 141]. This pathway has key roles in regulation of cellular growth and survival [142]. It has been reported that the PIK3CA mutations and amplification were a preliminary and prevalent occurrence among NMIBC patients which were correlated with reduced tumor relapse. In low-grade tumors there was also a correlation between PIK3CA and FGFR3 mutations. Moreover, patients with wt PIK3CA and FGFR3 mutated tumors had significantly higher recurrence rates. An exceptionally high rate of PIK3CA mutations and gene amplifications were particularly found in T1 and T2 tumors. Mutations and amplifications in PIK3CA induced AKT function [77]. PI3K can be activated by FGFR or ERBB through the binding of RAS to PIK3CA. It has been shown that the majority of UCC cases had mutations in PIK3CA, FGFR3, HRAS, KRAS, BRAF, and AKT1 genes. Mutations were significantly more frequent in FGFR3, PIK3CA, and FGFR3. The frequencies of mutations were negatively associated with grade. FGFR3mut and FGFR3mut- PIK3CAmut genotypes were correlated with low grade tumors, while the KRASmut- PIK3CAmut- and AKT1mut were observed in high-grade tumors [78]. Another study has been shown that the majority of patients with low-grade NMI-BC had a mutation in KRAS, NRAS, HRAS, PIK3CA, and FGFR3 genes. Therefore, mutational analysis of these genes along with regular cystoscopic examinations can be an efficient method of following-up among patients with grade 1–2 NMI-BC [79]. It has been observed that there was significant correlations between mutated FGFR3 and lower tumor stage/grade [80]. BCa patients with mutated FGFR3 had higher rate of vascularization in comparison with wild-type FGFR3. FVIII up regulation was the only angiogenic factor associated with mutated FGFR3. The T1 MIBC patients with mutated FGFR3 had significantly higher risk of recurrence compared with wild-type FGFR3 carriers. FGFR3 was also a poor prognostic factor and promoted tumor angiogenesis. Therefore, FGFR3-targeted therapies can be effective in reducing tumor angiogenesis [81]. Most of bladder tumors (75–80%) are papillary noninvasive (pTa) or superficially invasive (pT1) urothelial tumors, whereas the others (20–25%) are muscle-invasive (pT2). FGFR3 mutations are more common in pTa bladder tumors [82, 143, 144], less regular in pT1G3 tumors [145, 146], and rare in carcinoma in situ (pTis) [82, 146]. The BCa patients with a FGFR3 mutation appeared to have a better outcome compared with mutation free patients [144]. It has been reported that the FGFR3 and CK20 were efficient prognostic factors of pTa bladder tumors, since it can distinguish the differentiated tumors with FGFR3 mutations. The FGFR3 mutation with a normal CK20 expression pattern was observed in low-grade non-invasive papillary tumors [83]. It has been observed that the FGFR3 mutations were significantly more common in pTa BCa tumors. The patients with mutant FGFR3 had significantly lower death rate. The FGFR3 mutations can precisely determine patients with invasive tumor who were at lower risk of progression. The carriers of FGFR3 mutations had better prognosis [84]. Another study has been shown that the prevalence of FGFR3 mutation was significantly higher in pTa tumors compared with carcinoma in situ (CIS), pT1, and pT2–4 among a sub population of BCa patients. There were also significant correlations between FGFR3 mutations and low grade tumors [82]. It has been reported that the FGFR3 up regulation was more common in well-differentiated in comparison with poorly-differentiated tumors and in low stage tumors in comparison with advance stage tumors among BCa patients. There were significant associations between loss of FGFR3, stage, and grade [85]. A negative association was also reported between the levels of FGFR3 expression and grade. There was also significant inverse correlation between CDKN2A/p16 and FGFR3 expressions. CDKN2A/p16 up regulation and FGFR3 down regulation were significantly associated with poor progression-free survival [86]. MAP kinase, STAT1, PI3K-AKT, and PLCγ are important signaling pathways to mediate the FGFR3 functions [147–149]. It has been shown that there was a declining trend of FGFR3 expression from pTa toward pT3 cases in a sample of BCa cases. FGFR3 was also positive in 45, 26, and 30% of G1, G2 and G3 cases, respectively. Moreover, there was association between mutation and FGFR3 over expression which were frequent in primary stage (pTa and pT1) and low grade (G1 and G2) BCa tumors [87]. It has been shown that there was FGFR3 up regulation in a sample of BCa tumor tissues compared with normal mucosa. Tumor size was also correlated with FGFR3 expression. Moreover, the up regulations of FGFR3, PI3Kp110, PI3KClassIII, and AKT were also correlated with recurrence free survival among T1 BCa patients [88]. ERCC1 over expression and FGFR3 mutation were associated with a better response to the non-adjuvant chemotherapy in a group of MIBC patients [89]. It has been shown that the mRNA levels of FGFR1 and CK20 were significantly higher in BCa tissues in comparison with normal margins. The invasive tumors had significantly higher levels of FGFR1 and CK20 expressions compared with non-invasive tumors. FGFR1 or CK20 were sensitive markers to separate the invasive and non-invasive tumors. The levels of FGFR1 and CK20 expressions were significantly higher in invasive tumors (pT2-pT4) compared with non-invasive tumors (pTis, pTa, and pT1). The expression levels of FGFR1 and CK20 were also correlated with stage and grade [90].

### RTK classes of VIII, IX, and XIII

Tumor is limited to the mucosal layer in almost 70% of the new BCa cases. *The rest of patients have an advanced tumor stages with local lymph node involvement, muscles invasion, and distant metastases at the time of diagnosis. About 50% of the MIBC patients* does not respond to chemotherapy, radiotherapy, or surgical resection which results in a survival time lower than 5 years [116, 150]. The gold standard treatment option for BCa patients with distant metastases is the Platinum-based chemotherapy which has a 15% of 5-year survival rate and a median survival of 15 months [151]. MET is a cell surface RTK mainly produced in epithelial cells. MET signaling is critical for normal cellular development and homeostasis; however, it has also been shown to be involved in invasive tumors and distant metastasis [152]. It has been reported that the urinary MET levels could be an efficient marker of differentiating between BCa patients and healthy subjects, and also differentiating between MIBC and NMIBC patients [91]. Although, pharmaceutical inhibition of the RTK pathway function using Gefitinib had modest outcomes, it remains the gold standard treatment for BCa patients [153, 154]. The activation of c-MET by hepatocyte growth factor (HGF) worsen the malignant features of tumor cells which results in a higher rate of cells motility, proliferation, metastasis, and invasion [155]. Activation of c-MET induces other signaling proteins such as GRB2, GAB1, SHC, PLC1, and PI3-K [156]. Microarray analysis on RTK indicated that the PDGFR and AXL have interaction with c-MET [157]. It has been reported that the lack of c-MET expression renders less aggressiveness and more Cisplatin response in BCa. In contrast, c-MET up regulation had a significant association with worse clinical outcome and shorter overall survival among MIBC patients. The PDGFRL up regulation was also significantly correlated with a poorer prognosis. Moreover, NMIBC patients had an increased levels of AXL and PDGFR expressions compared with MIBC patients [92]. Recepteur d’Origine Nantais (RON) is a particular receptor tyrosine-kinase in the MET family [158]. It has been shown that there were correlations between RON or MET expressions and tumor aggressiveness and reduced survival. RON up regulation promoted the cell proliferation and migration. RON expression was also directly correlated with grade, size, and tumor stage among BCa patients. Moreover, RON/MET expression was correlated with reduced overall survival [93]. The Eph receptor is belonged to the RTK family that is regulated by ephrin ligands. Eph-ephrin interaction is associated with cell migration and neoplastic transformation [159]. It has been reported that there was a significant EphA2 up regulation in a sample of urothelial tumors compared with normal tissues. The levels of EphA2 expression was also significantly correlated with tumor stage. Moreover, there was a converse correlation between E-cadherin and EphA2 expressions in advanced tumor stages [94]. EphB4 is a member of the Eph receptors which has key functions in angiogenesis, neural development, and pattern formation [160–163]. EphB4 and its specific ligand, EphrinB2, are both transmembrane proteins that are typically expressed on venous and arterial endothelium, respectively. Deregulation of EphB4 has been demonstrated in various tumors of breast, prostate, and lung [164–167]. Activation of EphB4 regulates cell attachment and migration [168–171]. Frequent EphB4 up regulation was reported among a sample of BCa patients. While, majority of tumor tissues showed a high expression of EphB4, normal urothelial cells displayed very little or lack of EphB4 expression. P53 is a regulator of EphB4 via MAPK and PI3K signaling pathways. EphB4 was up regulated by PI3K/AKT pathway. The EphB4 suppression also reduced tumor cells invasion which can be due to MMP9 down regulation. Moreover, they observed BCL-XL down regulation following the EphB4 knockdown in BCa cells. Therefore, EphB4 suppression reduced tumor progression and increased apoptosis [95]. Discoidin domain receptors (DDRs) are a class of RTKs which are activated by collagens. DDR1 can be activated by most collagen types, whereas DDR2 can be activated only by type I and III collagens [172]. The collagen-DDR1 binding increases the self-renewal and migration of non-cancerous cells [173, 174]. A high level of DDR1 in solid malignant tumors was associated with poor prognosis [175]. It has been reported that there was a significant increased levels of DDR1 expression in BCa tissues which was associated with poor prognosis. DDR1 activation also increased tumor invasion through ZEB1 and SLUG up regulations in bladder tumor cells [96]. DDR2 promotes the EMT through the ECM blockade. The activated DDR2 becomes docking sites for adaptor proteins, resulting in the MMP2 up-regulation [176]. It has been observed that there was a significant DDR2 up regulation in a sample of urothelial carcinoma patients which was associated with poor prognosis, infiltrative pattern, higher grade, advanced T stage, and metastatic status [97].

### Other tyrosine-kinase receptors

Brain-derived neurotrophic factor (BDNF) is a member of the neurotrophin family of growth factors associated with neural differentiation and survival through binding to the Tropomyosin receptor kinase B (TrKB) [177]. BDNF/TrKB pathway has been reported to be involved in different solid cancers such as lung, prostate, and pancreatic ducts [178–180]. It has been observed that there were increased levels of BDNF and TrKB expressions among a sub population of transitional cell carcinomas (TCC) patients compared with healthy subjects. However, differences were significant in grade 3 TCC for BDNF expression, and in grade 1 and 3 TCC for TrKB expression. Therefore, TrKB and/or BDNF up regulation can be introduced as efficient markers of *early detection among BCa patients* [98]*.* C-KIT is a tyrosine-kinase receptor involving in carcinogenesis and hematopoiesis [181, 182]. The c-KIT up regulation has been reported in various cancers such as gastrointestinal, lung, and breast cancers [183–185]. Since, c-KIT up regulation has been observed in a marked population of a sample of small cell BCa patients, c-KIT targeted therapies can be an effective therapeutic method among these patients [99]. The c-KIT has key developmental functions and mainly expressed in melanocytes, interstitial cells of Cajal, erythroid and mast cell lineages [186–188]. The prevalence of c-KIT mutation and its expression levels were assessed in a sub population of bladder SCC patients which showed c-KIT up regulation in majority of cases. Bilharzial ova positive patients also showed a significantly higher expression levels of c-KIT compared with Bilharzial ova negative cases [100].

### Non-receptor tyrosine-kinases

Feline Sarcoma (FES) and FES-related protein (FER) are a separate sub-family of non-receptor tyrosine-kinases [189]. Down regulation of FES represses the progression of renal cell carcinoma cells [190]. It was also shown that the increased FES expression was associated with a more aggressive tumor and shorter recurrence-free survival following surgical resection [191]. The FES was significantly down regulated in tumor cells compared with normal urothelial cells. A positive association was also observed between FES expression level and tumor cells invasion in patients with high-grade tumors. Moreover, FES up regulation was determined as a negative prognostic predictor of metastasis after radical surgery in patients with high-grade malignancies [101]. Dual-specificity tyrosine phosphorylation-regulated kinases (DYRKs) constitute a subfamily of protein kinases which have the ability to phosphorylate aromatic (tyrosine) besides aliphatic (serine and threonine) residues [192–194]. DYRKs are involved in regulation of cellular proliferation, differentiation, and survival [195, 196]. DYRK2 stimulates cell apoptosis following DNA damages by p53 phosphorylation during genotoxic stress [197]. It has been reported that there was a significant correlation between DYRK2 over expression and higher disease-specific survival among chemotherapeutic-treated T1 high-grade and T2 BCa patients. Therefore, assessing the levels of DYRK2 could be a predictive factor to detect patients with T1 high-grade and T2 BCa that will probably show a good response to neoadjuvant chemotherapeutic treatment [102]. Epithelial and endothelial tyrosine-kinase (ETK) is a family of non-receptor tyrosine-kinases which can be activated by cytokines, hormones, growth factors, and ECM [198]. ETK is involved in regulation of cell proliferation, differentiation, motility, and survival. It has been reported that the ETK levels rises gradually during the BCa progression. There were also significant correlations between ETK up regulation, higher tumor grade, and poor prognosis. Suppression of ETK in bladder tumors reduced activity of AKT and STAT3. Therefore, the ETK over expression can be the reason of increased AKT and STAT3 activity in bladder tumors [103].

## Conclusions

It was observed that the class I and V of RTKs were the most reported tyrosine-kinases among BCa patients in the world. This review highlights the importance of tyrosine-kinases as critical markers in early detection and therapeutic purposes among BCa patients and clarifies the molecular biology of tyrosine-kinases during BCa progression and metastasis.

## Data Availability

The datasets used and/or analyzed during the current study are available from the corresponding author on reasonable request.

## Abbreviations

BCa: Bladder cancer
US: United States
NMIBC: Non-muscle-invasive bladder cancer
MIBC: Muscle-invasive bladder cancer
TKs: Tyrosine-kinases
EGFR: Epidermal growth factor receptor
PDGFR: Platelet-derived growth factor receptor
VEGFR: Vascular endothelial growth factor receptor
FGFR: Fibroblast growth factor receptor
HGFR: *Hepatocyte growth factor receptor*
TKIs: Tyrosine-kinase inhibitors
RTKs: Receptors of tyrosine-kinases
FASN: Fat synthase
UUTUC: Upper urinary tract urothelial carcinoma
HRG: Heregulin
MPUC: Micropapillary urothelial carcinoma
IGF1R: Insulin-like growth factor-1 receptor
bFGF: Basic Fibroblast Growth Factor
CIS: Carcinoma in situ
HGF: Hepatocyte growth factor
RON: Recepteur d’Origine Nantais
BDNF: Brain-derived neurotrophic factor
TCC: Transitional cell carcinomas
FES: Feline Sarcoma
FER: FES-related protein
ETK: Epithelial and endothelial tyrosine-kinase
PI3K: Phosphatidylinositol 3-kinase
DDRs: Discoidin domain receptors
